# CD44 positive and sorafenib insensitive hepatocellular carcinomas respond to the ATP-competitive mTOR inhibitor INK128

**DOI:** 10.18632/oncotarget.25430

**Published:** 2018-05-25

**Authors:** Mohamed Badawi, Jihye Kim, Anees Dauki, Dhruvitkumar Sutaria, Tasneem Motiwala, Ryan Reyes, Nissar Wani, Shamalatha Kolli, Jinmai Jiang, Christopher C. Coss, Samson T. Jacob, Mitch A. Phelps, Thomas D. Schmittgen

**Affiliations:** ^1^ College of Pharmacy, College of Medicine, The Ohio State University, Columbus, OH, USA; ^2^ Department of Molecular and Cellular Biochemistry, College of Medicine, The Ohio State University, Columbus, OH, USA; ^3^ College of Pharmacy, University of Florida, Gainesville, FL, USA; ^4^ Comprehensive Cancer Center, The Ohio State University, Columbus, OH, USA

**Keywords:** TAK228, MLN0128, sapanisertib, pharmacokinetics

## Abstract

The mTOR pathway is activated in about 50% of patients with hepatocellular carcinoma (HCC). In an effort to identify new pathways and compounds to treat advanced HCC, we considered the ATP-competitive mTOR inhibitor INK128. ATP-competitive mTOR inhibitors attenuate both mTORC1 and mTORC2. INK128 was evaluated in sorafenib sensitive and insensitive HCC cell lines, CD44_low_ and CD44_high_ HCC and those cell lines with acquired sorafenib resistance. CD44 was significantly increased in Huh7 cells made resistant to sorafenib. Forced expression of CD44 enhanced cellular proliferation and migration, and rendered the cells more sensitive to the anti-proliferative effects of INK128. INK128 suppressed CD44 expression in HCC cells while allosteric mTOR inhibitors did not. CD44 inhibition correlated with 4EBP1 phosphorylation status. INK128 showed better anti-proliferative and anti-migration effects on the mesenchymal-like HCC cells, CD44_high_ HCC cells compared to the allosteric mTOR inhibitor everolimus. Moreover, a combination of INK128 and sorafenib showed improved anti-proliferative effects in CD44_high_ HCC cells. INK128 was efficacious at reducing tumor growth in CD44_high_ SK-Hep1 xenografts in mice when given as monotherapy or in combination with sorafenib. Since the clinical response to sorafenib is highly variable, our findings suggest that ATP-competitive mTOR inhibitors may be effective in treating advanced, CD44-expressing HCC patients who are insensitive to sorafenib.

## INTRODUCTION

Hepatocellular carcinoma (HCC) is the fifth most prevalent cancer and has the second highest mortality rate of all cancers worldwide [1]. HCC is predicted to become the third most lethal cancer in the USA by 2030 [2]. Reasons for the rise in the incidence of HCC include increased Hepatitis C and Hepatitis B infection, as well as increased rates of obesity resulting in non-alcoholic steatohepatitis [3]. Percutaneous ablation is an option in patients who are afflicted with early HCC and who are not candidates for resection or transplantation [4]. Transarterial chemoembolization has been effective in patients with intermediate stage HCC [5]. HCC patients that are diagnosed with advanced disease or whose cancer recurs following regional therapy have a dismal prognosis. The multikinase inhibitor sorafenib is currently the standard of care for advanced HCC [6]. Sorafenib targets RAF/MEK/ERK pathway by inhibiting Raf serine/threonine kinase and cell surface receptor tyrosine kinases including vascular endothelial growth factor receptor and platelet-derived growth factor receptor [7]. Sorafenib treatment showed clinically modest improvement in overall survival [8], and great variability in response between patients [6, 8].

The PI3K/AKT/mTOR pathway is an important intracellular signaling pathway which is responsible for cell proliferation, growth, survival, protein synthesis and glucose metabolism [9]. PI3K/AKT/mTOR signaling is abnormally activated in HCC [10–12]. Long-term exposure to sorafenib resulted in the activation of PI3K/AKT/mTOR signaling in sorafenib resistant Huh7 cells compared to parental Huh7 cells [13]. A multicenter, randomized phase III clinical trial aiming to overcome sorafenib intolerance was conducted with the allosteric mTOR inhibitor, everolimus [14]. Everolimus failed to show improved overall survival in patients with advanced HCC who were resistant or intolerant to sorafenib, possibly due to everolimus-induced feedback activation of AKT following selective mTORC1 complex inhibition [15]. Unlike the rapalogs, the ATP competitive mTOR inhibitors attenuate both mTORC1 and mTORC2 [16–18]. Very recently mTORC2 was reported to promote tumorigenesis in HCC via increased lipogenesis [19]. This highlights the importance of blocking both mTORC1 and mTORC2 when considering therapies for HCC. Two ATP competitive inhibitors, AZD2014 and OSI-027 were shown to be effective in HCC [20, 21]. AZD2014 was more effective at blocking mTORC1 than rapamycin [21]. OSI-027 reduced HCC proliferation, synergized with doxorubicin and inhibited phosphorylation of the mTORC1 and mTORC2 downstream effectors, 4EBP1, p70S6K and AKT [20].

CD44, hyaluronic acid receptor, plays important roles in cell-cell interactions, cell adhesion, invasion/metastasis and is expressed on HCC tumor-initiating cells [22, 23]. A recent meta-analysis concluded that CD44 expression was associated with TNM stage in HCC and that positive CD44 expression was associated with a worse overall survival than CD44-negative expression [24]. CD44 expression also increased in sorafenib resistant HCC cell lines [25]. Interestingly, the mTOR ATP site inhibitors PP242 or INK128 reduced cell invasion and metastasis of prostate cancer cells [26] and this reduction correlated with a decrease in CD44. CD44 inhibition was achieved in these cells by blocking p70S6K and 4EBP1. We have previously shown that inhibiting the PI3K-AKT-mTOR pathway with PP242 reduced CD44 protein in SNU-423 and SNU-449 cells without altering CD44 mRNA levels [27]. We evaluated here the second generation mTOR ATP site inhibitors PP242 and INK128 in sorafenib sensitive and insensitive HCC cell lines, CD44_low_ and CD44_high_ HCC and those cell lines with acquired sorafenib resistance. We demonstrate that the more aggressive-sorafenib insensitive, CD44_high_ HCC respond better to mTOR ATP site inhibitors than their counterparts. Our data suggest INK128 as an alternative therapeutic to be used as a single agent or in combination with sorafenib for patients with CD44-positive HCC. It also highlights the potential use of CD44 as a biomarker of response to therapy with ATP-competitive mTOR inhibitors.

## RESULTS

### INK128 shows enhanced anti-proliferative effects compared to allosteric mTOR inhibitors

**Figure 1:**
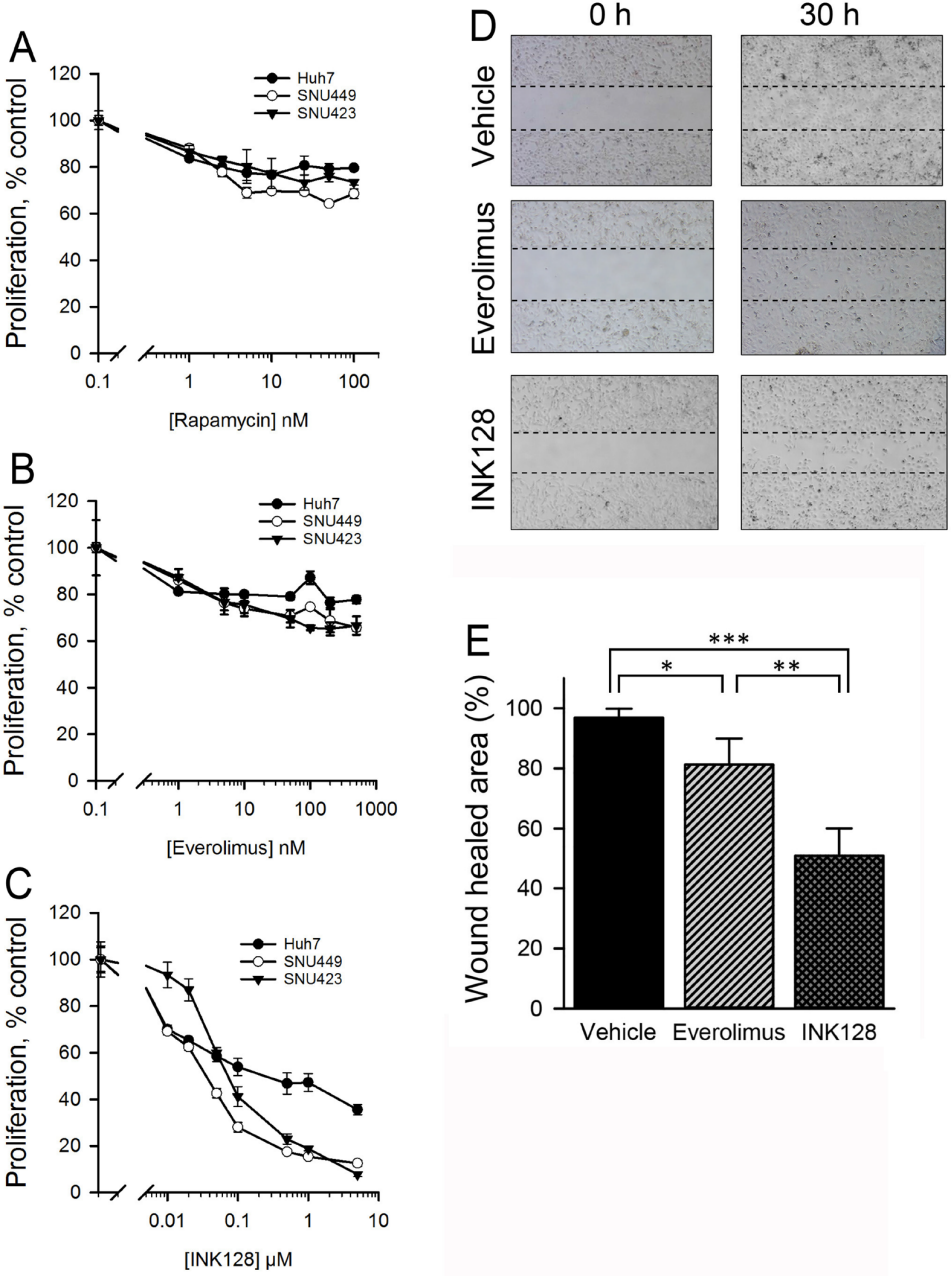
INK128, but not allosteric mTOR inhibitors, show enhanced activity in SNU423 and SNU449 HCC cells The HCC cell lines SNU423, SNU449 and Huh7 were treated with the allosteric mTOR inhibitors (**A**) rapamycin, (**B**) everolimus or (**C**) the ATP-competitive mTOR inhibitor INK128 for 48 h. Cell proliferation was determined by a WST-1 assay. (**D**) SNU423 cells were treated with vehicle control or 500 nM everolimus or INK128. Dashed lines represent the starting point of the *in vitro* migration assay at time 0. At 30 h after cell migration, the gap open areas were photographed and measured (**E**). ^*^p < 0.05, ^**^p < 0.01, ^***^p < 0.001. Single replicates from at least duplicate experiments.

The anti-proliferative effect of rapamycin, everolimus, PP242 or INK128 was evaluated in three different HCC cell lines (SNU423, SNU449 and Huh7). Rapamycin and everolimus failed to reduce proliferation below 40%, even at concentrations of 5000 ng/ml and 5000 nM, respectively (Figure 1A–1B). The ATP-competitive inhibitors INK128 (Figure 1C) and PP242 (Supplementary Figure 1) exhibited better anti-proliferative activity than rapamycin and everolimus in SNU423 and SNU449 cells. Interestingly, the CD44_low_ cell line Huh7 responded poorly to all of the tested inhibitors. We next compared the ability of everolimus and INK128 to inhibit *in vitro* cell migration. SNU423 cells were treated with 500 nM everolimus or INK128. Everolimus and INK128 treated cells decreased cell movement by 20% and 50% compared to vehicle control, respectively (Figure 1D, 1E) indicating that INK128 showed improved anti-migration effects compared to everolimus in SNU423 cells.

### ATP competitive inhibitors are more effective than rapalogs at reducing proliferation and CD44 and vimentin expression in CD44_high_ HCC cells

**Figure 2:**
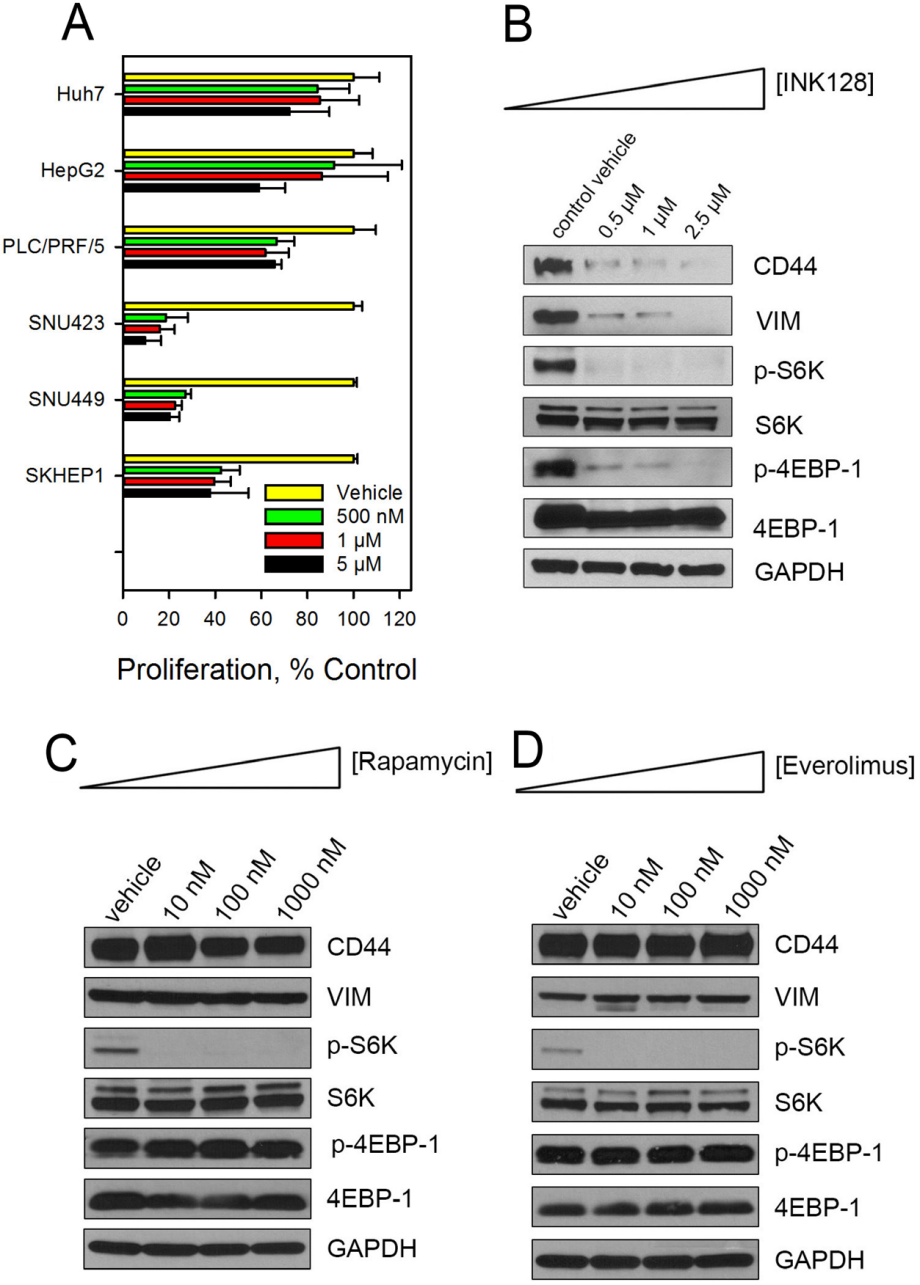
INK128 selectively kills CD44_high_ HCC cells (**A**) CD44_low_ cells (Huh7, PLC/PRF/5 and HepG2) and CD44_high_ (SNU423, SNU449, SK-Hep-1) HCC cells were treated with increasing concentrations of INK128 for 72 h. Cell proliferation was determined using a WST-1 assay. CD44_high_ SNU423 cells were treated with increasing concentrations of (**B**) INK128, (**C**) rapamycin or (**D**) everolimus for 48 h. CD44, vimentin and downstream effectors of mTOR pathway were evaluated by immunoblotting. Single replicates from at least duplicate experiments.

The data presented in Figure 1C and Supplementary Figure 1 show a differential activity of the ATP competitive inhibitors in SNU423 and SNU449 cells compared to Huh7. SNU423 and SNU449 represent aggressive, more mesenchymal-like HCC cell lines compared to Huh7 which displays more of an epithelial phenotype [28, 29]. We previously reported that SNU423 and SNU449 express much higher protein level expression of the hyaluronic acid receptor CD44 than does Huh7 [30]. To further evaluate the role of the ATP competitive inhibitors in HCC as a function of CD44 expression, we selected three CD44_high_ (SNU423, SNU449 and Sk-Hep-1) and three CD44_low_ (Huh7, PLC/PRF/5, HepG2) cell lines (Supplementary Figure 2). INK128 was more effective at reducing proliferation in the three CD44_high_ HCC cells compared to the CD44_low_ cells (Figure 2A). We have previously reported the inhibition of CD44 in SNU423 and SNU449 cells by PP242 [27]. To investigate the possible mechanisms for the differential response of cells to allosteric and ATP competitive mTOR inhibitors, especially in the context of improved sensitivity of CD44_high_ HCC cells to INK128 treatment, various proteins associated with the mTOR pathway and aggressive HCC were measured in CD44_high_ cells treated with rapamycin, everolimus or INK128. In contrast to the ATP competitive mTOR inhibitor, INK128 (Figure 2B), rapamycin and everolimus did not alter CD44 and vimentin protein expression in SNU423 cells (Figure 2C, 2D) or SNU449 (Supplementary Figure 3) cells. S6K and 4EBP1, the main downstream targets of the mTOR signaling pathway, were next evaluated. S6K and 4EBP1 control protein synthesis by regulating mRNA translation initiation and progression [31]. Rapamycin and everolimus blocked phosphorylation of S6K but did not inhibit phosphorylation of 4EBP1 (Figure 2C, 2D, Supplementary Figure 3). INK128 (Figure 2B), however, inhibited phosphorylation of both S6K and 4EBP1. This inhibition, likely contributed to the reduced CD44 protein expression in HCC cells. We further investigated whether INK128 altered the expression of CD44 or vimentin transcriptionally or post-translationally. There was no change in the CD44 or vimentin mRNA in SNU423 cells treated with INK128 (Supplementary Figure 4) even though immunoblotting revealed a great reduction in CD44 and vimentin protein expression by the ATP competitive mTOR inhibitors (Figure 2B). These data demonstrate that expression of CD44 and vimentin correlates with 4EBP1 phosphorylation status in CD44_high_ HCC cells and suggests that inhibiting phosphorylation of 4EBP1 by competitive mTOR inhibitors is a contributing factor to attenuating CD44 expression in HCC cells.

### Response to INK128 directly correlates with CD44 levels

**Figure 3:**
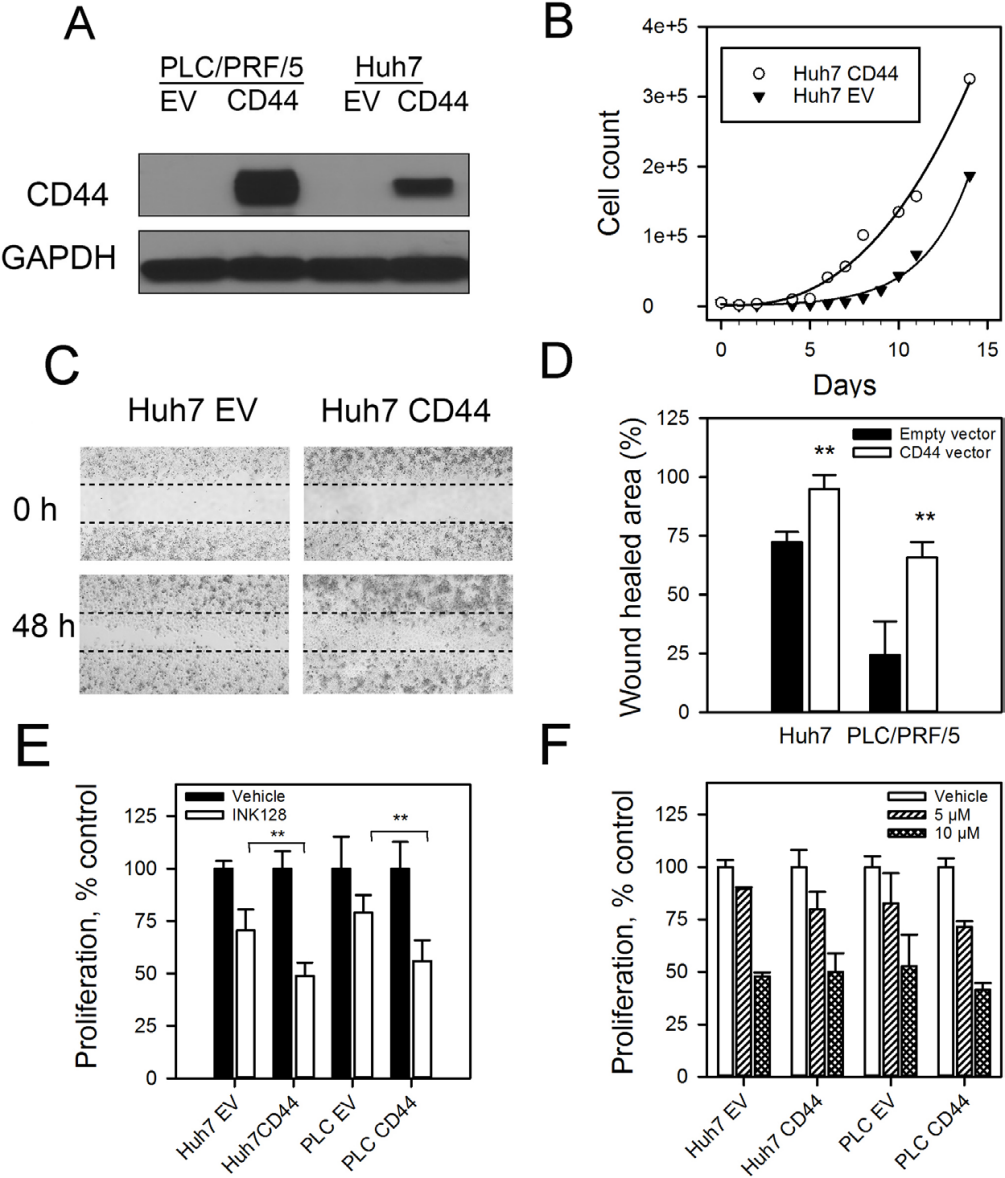
CD44 overexpressing cells have a more aggressive phenotype and improved response to INK128 (**A**) PLC/PRF/5 and Huh7 cells were stably transfected with CD44 expressing vector or empty vector (EV) control. CD44 and GAPDH levels were measured by immunoblotting. (**B**) Cell growth curves of Huh7_CD44_ and Huh7_EV_ cells. (**C**) *In vitro* migration of Huh7_CD44_ and Huh7_EV_ cells. (**D**) *In vitro* migration of Huh7_CD44_ and Huh7_EV_ and PLC/PRF/5_CD44_ and PLC/PRF/5_EV_ cells. (**E**) Huh7_CD44_, Huh7_EV_, PLC/PRF/5_CD44_ and PLC/PRF/5_EV_ cells were treated with 500 nM INK128 or vehicle control for 72 h. Cell proliferation was measured by a WST-1 assay. (**F**) Huh7 and PLC/PRF/5 expressing CD44 or EV control cells were treated with sorafenib for 72 h and cell viability was determined using WST-1 reagent. ^**^p < 0.01, Student's *t*-test.

For further investigation, we used INK128 rather than PP242 as INK128 has enhanced drug-like properties compared to PP242 (e.g. improved oral bioavailability) and is currently being investigated in clinical trials for several cancers including breast, prostate, thyroid, lung and HCC (NCT02575339, clinicaltrials.gov). To further examine the relationship between CD44 expression and INK128 response, we stably overexpressed the CD44 protein in the CD44_low_ Huh7 and PLC/PRF/5 cells to generate Huh7_CD44_ and PLC/PRF/5_CD44_ cells (Figure 3A). We evaluated the effect of CD44 on *in vitro* cellular proliferation and migration. Huh7_CD44_ and PLC/PRF/5_CD44_ cells had improved proliferation (Figure 3B, Supplementary Figure 5A) and enhanced migration (Figure 3C, 3D, Supplementary Figure 5B) compared to those cells stably expressing the empty vector. We next treated Huh7_CD44_, PLC/PRF/5_CD44_, Huh7_EV_ and PLC/PRF/5_EV_ cells with INK128. Interestingly, the HCC cells stably expressing CD44 responded better to INK128 than did those HCC cells stably expressing the empty vector (Figure 3E).

### Sorafenib sensitivity in HCC cell lines

The antiproliferative effects of sorafenib in CD44_high_ and CD44_low_ HCC cells were evaluated. Fernando *et al*. reported that HCC cells that are mesenchymal-like and have high CD44 expression are refractory to sorafenib-induced cell death *in vitro* [32]. To further investigate the relationship between sorafenib, INK128 and CD44, we treated the CD44 expressing Huh7_CD44_ and PLC/PRF/5_CD44_ and their respective controls with sorafenib or INK128 for 72 h. Unlike INK128 (Figure 3E), there was no difference in sorafenib's ability to reduce proliferation as a function of CD44 protein (Figure 3F).

### Combination of INK128 and sorafenib

**Figure 4:**
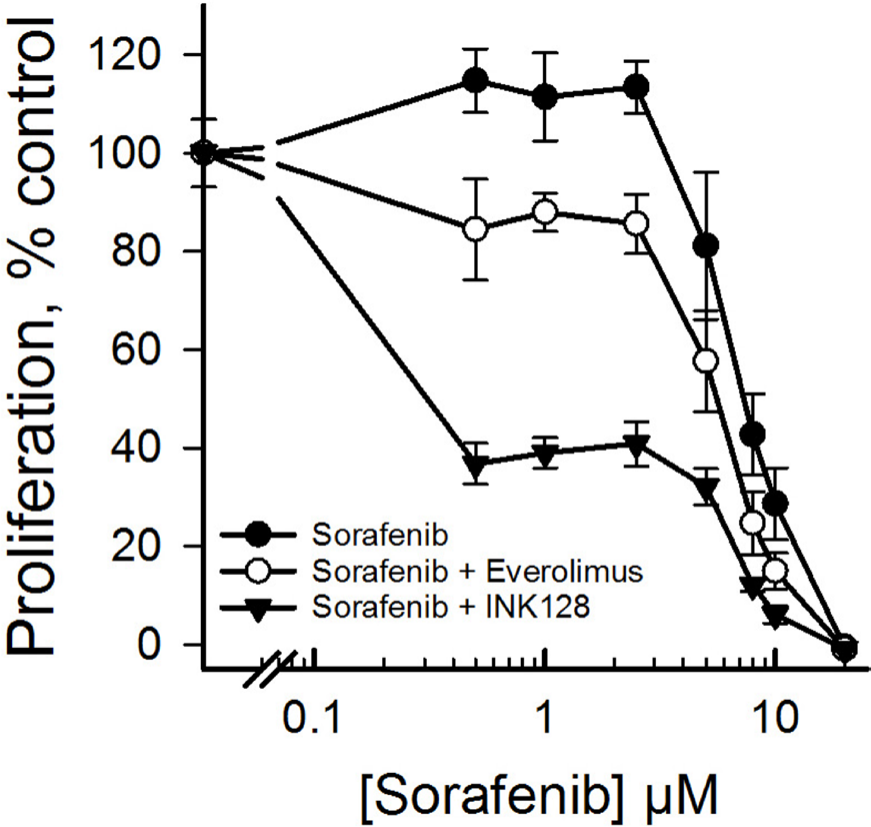
Improved antiproliferative effects of INK128 alone or in combination with sorafenib The CD44_high_ cells SNU423 cells were treated with sorafenib alone or in combination with everolimus or INK128 for 72 h. Cell proliferation was determined using a WST-1 assay. Single replicate from at least duplicate experiments.

The favorable anti-proliferative effects of INK128 in CD44_high_ HCC cells led us to test whether addition of this drug to the current standard of care for advanced HCC (sorafenib) has the potential to improve outcomes over that of sorafenib alone. We included everolimus in this test to determine whether the lack of response to everolumus can be enhanced in combination with sorafenib. CD44_high_ SNU423 cells were treated with increasing concentrations of sorafenib plus 100 nM INK128. While addition of INK128 to sorafenib resulted in a dramatic reduction in cell proliferation, the addition of everolimus did not have such a significant effect on proliferation of SNU423 cells, when compared to sorafenib alone (Figure 4).

Since combining sorafenib with INK128 showed favorable reduction in proliferation compared to everolimus (Figure 4), further combination studies were performed to understand the nature of the interaction between sorafenib and INK128. INK128 was combined with sorafenib at a fixed molar ratio (1:200 for SNU449 and 1:150 for SNU423 and Huh7). Combining INK128 and sorafenib resulted in an additive to synergistic effect in CD44_high_ SNU423 and SNU449 cells as evident by combination index values equal to or less than one (Table 1). However, this trend was not observed in CD44_low_ Huh7 cells, as the combination of both drugs did not result in enhanced antiproliferative effects.

**Table 1:** Combination indices for sorafenib and INK128

Cell line	INK128:Sorafenib	Combination Index (CI)	
		IC_75_	IC_90_
SNU449	1:200	0.614	0.506
SNU423	1:150	1.065	0.830
Huh7	1:150	1.496	3.231

### INK128 is effective in sorafenib resistant HCC

**Figure 5:**
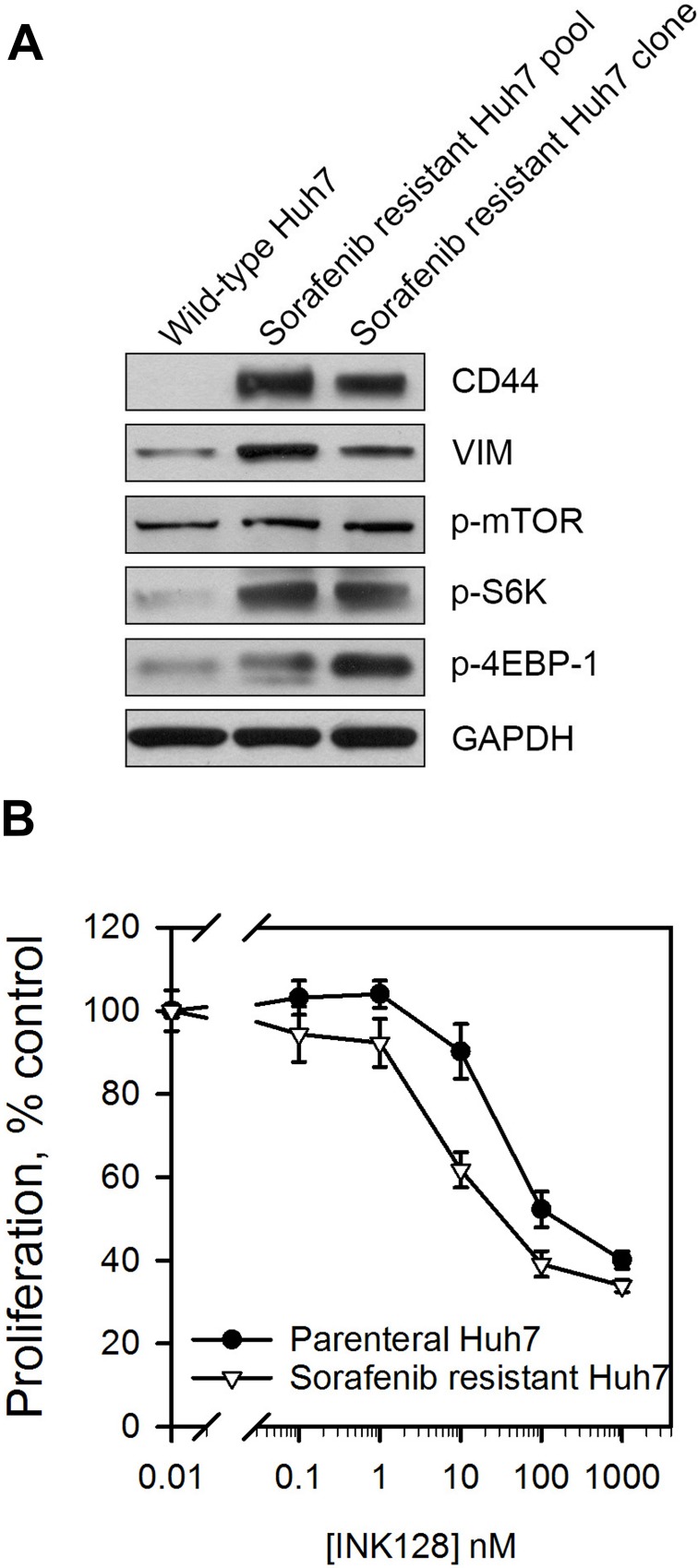
Sorafenib resistant cells display enhanced mTOR pathway activation and improved sensitivity to INK128 (**A**) CD44, vimentin and downstream effectors of the mTOR pathway were measured in wild-type Huh7 or Huh7 cells with acquired sorafenib resistance. (**B**) Sorafenib resistant Huh7 or parenteral cells were treated with INK128 for 72 h. Cell proliferation was measured by a WST-1 assay. Single replicates from at least duplicate experiments.

Next, we wanted to determine whether INK128 could be an effective treatment option for sorafenib resistant HCC. Since this inhibitor negatively regulates CD44 protein expression possibly through suppressing phosphorylation of 4EBP-1 in CD44_high_ HCC cells (Figure 2 and Supplementary Figure 3), we first evaluated the CD44 and mTOR pathways in HCC cells with acquired sorafenib resistance. CD44 and vimentin expression were increased in both the pool and clone of sorafenib resistant cells (Figure 5A). The increased phosphorylation of mTOR, S6K and 4EBP-1 (Figure 5A) demonstrated that mTOR pathway was activated in sorafenib resistant Huh7 cells. The anti-proliferative effects of INK128 were studied on HCC cells with acquired sorafenib resistance. INK128 showed greater anti-proliferative effects against sorafenib resistant Huh7 cells (CD44 expressing) compared to the wildtype CD44_low_ Huh7 cells (Figure 5B).

### INK128 pharmacokinetic study

**Figure 6:**
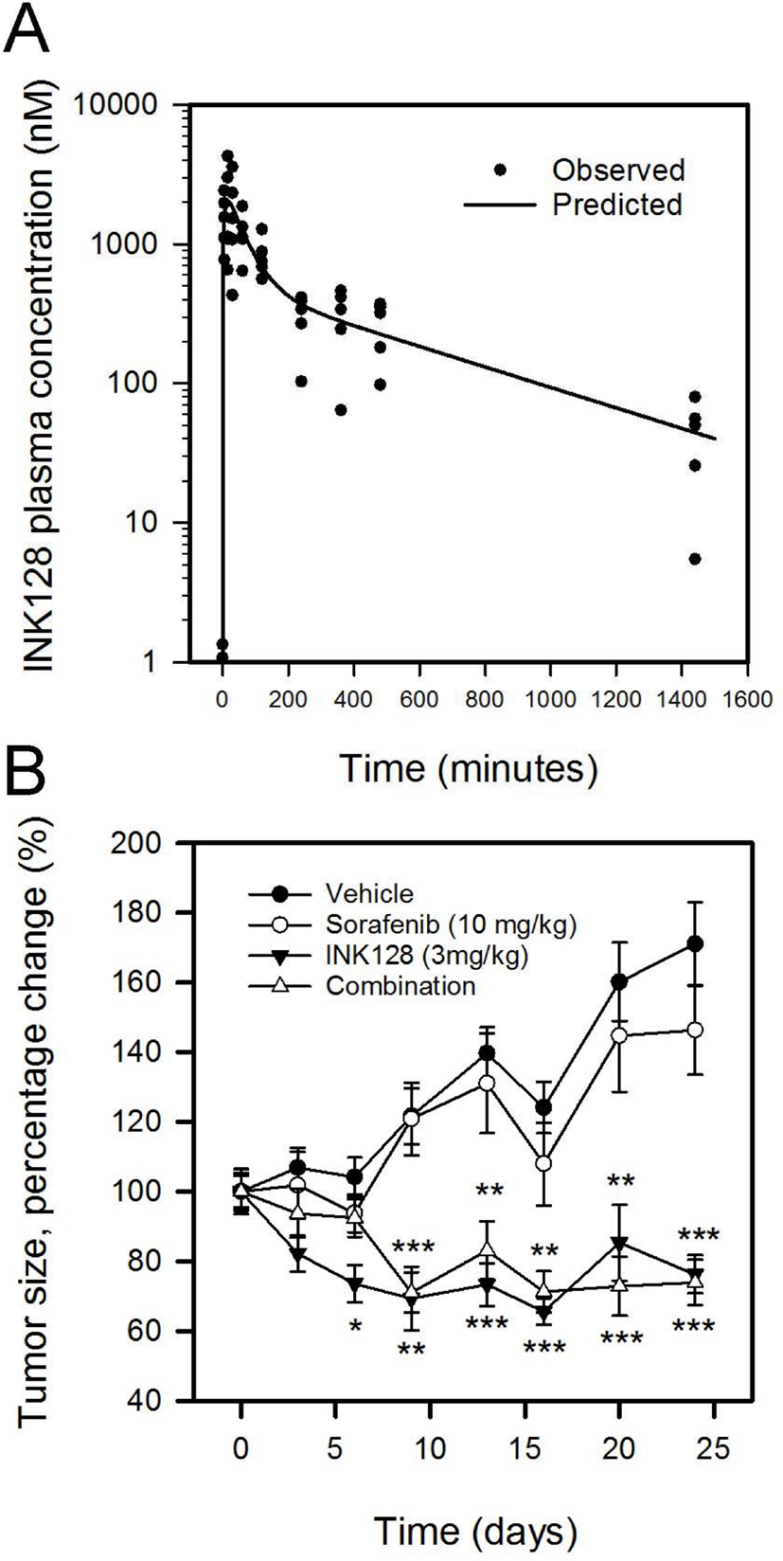
INK128 inhibits growth of SK-Hep1 xenografts in mice (**A**) Plasma pharmacokinetics of a single, oral dose of INK128 (3 mg/kg). Observed concentrations at specified time post dose are represented as dots. Line is a fit of the naïve pooled data. (**B**) Tumor volume measurements of SK-Hep1 subcutaneous xenografts in mice receiving vehicle, sorafenib, INK128 or combination treatments. Sorafenib was dosed daily at 10 mg/kg. INK128 was dosed every other day at 3 mg/kg. Data are presented as change from baseline tumor volume. ^*^P < 0.05, ^**^P < 0.01, ^***^P < 0.001, compared to vehicle control.

To characterize the *in vivo* pharmacokinetics of INK128 in mice, ICR mice were dosed with INK128 in 10% DMSO at a 3 mg/kg dose via oral gavage. Complete solubilization of the drug was confirmed by visual inspection and UV spectrophotometry. Figure 6A shows the concentration-time plots of the observed data, and the two-compartmental fit to the naïve pooled data. INK128 was readily absorbed orally, with detectable drug being observed at 5 min, and a maximum concentration (C_max_) of 2027 nM achieved at 15 min (t_max_) post dose. Elimination of the drug followed first order kinetics and there was detectable concentrations of INK128 (24.6 nM) 24 h post dose. Table 2 summarizes the non-compartmental pharmacokinetic parameters of INK128.

**Table 2:** Pharmacokinetic parameters of INK128

Parameter	Estimate
T_max_ (min)	15
C_max_ (nM)	2026.8
T_1/2_ (min)	108.7
CL (L/min)	0.000582
AUC_last_ (min*nM)	443029.4

### INK128 efficacy study

In order to study the ability of INK128 to prevent tumor progression *in vivo*, an efficacy study was carried out. Athymic nude mice bearing SK-Hep1 subcutaneous xenografts, were randomized and treated with one of the following; vehicle, sorafenib (10 mg/kg), INK128 (3 mg/kg), and combination of the two. SK-Hep-1 xenografts were used rather than SNU-423 or SNU-449 as we were unable to establish xenografts using the SNU cell lines. SK-Hep-1 cells express comparable levels of CD44 as the SNU cell lines (Supplementary Figure 2) and SK-Hep-1 responded to INK128 *in vitro* similar to the SNU cell lines (Figure 2A). Tumor size increased by 71% and 46% compared to baseline in vehicle and sorafenib groups, respectively (Figure 6B). On the other hand, INK128 alone or in combination with sorafenib resulted in inhibition of new tumor growth and moderate shrinkage of the tumor as seen by a 24% and a 26% reduction in tumor volume, respectively (Figure 6B). There was no significant variability in body weights across the groups, as body weight fluctuated between 98.5% and 115% from baseline body weight.

## DISCUSSION

We report here that the ATP competitive mTOR inhibitor INK128 negatively regulates CD44 translation in HCC possibly by blocking phosphorylation of 4EBP1, a key downstream protein in the mTOR pathway. We report that CD44 is expressed in sorafenib insensitive and sorafenib resistant cells [25, 32] and that the PI3K/AKT/mTOR signaling pathway is activated in sorafenib resistant Huh7 cells [13]. CD44 is a major cell matrix-associated protein whose expression has been correlated with enhanced tumor aggressiveness, metastasis and lack of response to a variety of therapies [24, 25]. We show that INK128 has potent anti-proliferative and anti-migratory activities against CD44-expressing sorafenib insensitive, mesenchymal-like HCC cells. These potent anti-proliferative effects were not observed in cells with low or no CD44 expression.

We confirmed that CD44 is increased and several components of mTOR pathway are activated in sorafenib resistant Huh7 cells (Figure 5A). The ATP-competitive mTOR inhibitors suppress CD44 and are effective in sorafenib insensitive and CD44 expressing HCC cell lines (Figure 1, Supplementary Figure 1). The allosteric mTOR inhibitors rapamycin and everolimus blocked phosphorylation of S6K but not 4EBP1 (Figure 2, Supplementary Figure 3). It has been reported that rapamycin inhibited phosphorylation of 4EBP1 initially, however phosphorylated 4EBP1 recovered within 6 h of treatment [33]. We also failed to observe inhibition of phosphorylation of 4EBP1 at 48 h treatment of the allosteric mTOR inhibitors. As we previously reported for PP242 [27] and report here for INK128 (Figure 2), the ATP-competitive mTOR inhibitors blocked phosphorylation of both S6K and 4EBP1. These agents successfully suppressed CD44 expression in sorafenib insensitive, mesenchymal-like HCC cells while rapamycin and its analog, everolimus did not (Figure 2, Supplementary Figure 3).

CD44 expression has been reported to contribute to a lack of response to sorafenib-induced apoptosis. However, the sole expression of CD44 did not result in sorafenib insensitivity and had to be accompanied with TGF-β activation [32]. We report similar findings, where stable expression of CD44 in the epithelial like cell lines Huh7 and PLC/PRF/5 did not induce sorafenib resistance (Figure 3F) even though cellular proliferation and migration were enhanced (Figure 3 and Supplementary Figure 5). Interestingly, CD44 overexpression resulted in an increased sensitivity of the cells to the anti-proliferative effects of INK128 (Figure 3E). Further examination of Huh7 cells with acquired sorafenib resistance revealed activation of the mTOR pathway along with CD44 overexpression (Figure 5A). This confirms that CD44 alone does not induce resistance, and that mTOR activation is the main driver of sorafenib resistance in this cell model.

Everolimus has failed a phase 3 clinical trial in advanced HCC patients who progressed during or after sorafenib therapy [14]. Since INK128 was effective in CD44 positive and sorafenib resistant cell lines, we explored the possibility of combining INK128 and sorafenib to achieve greater anti-cancer effects. The INK128/sorafenib combination showed an additive to synergistic effect in CD44_high_ cell lines (Table 1), although the main antiproliferative effect was achieved by INK128. These data suggest that INK128 alone or in combination with sorafenib may be effective in CD44 positive HCC. Interestingly, INK128 was not effective either alone (Figure 2A) or in combination (Table 1) in CD44_low_ cells. In these cases, sorafenib was the more effective drug, possibly due to low mTOR activation in these cells.

INK128 is under investigation in clinical trials for treatment of different types of cancer including renal cell carcinoma, glioblastoma, hepatocellular carcinoma and breast cancer (clinicaltrials.gov). We characterized the pharmacokinetics of INK128 in mice using a previously published effective dose of 3 mg/kg [34]. These data are beneficial in guiding future investigations in mouse models, and understanding toxicities associated with INK128. Following a 3 mg/kg dose, maximum drug concentration was achieved within 15 min, followed by active concentration for up to 24 h. Drug concentrations were greater than 100 nM for approximately 16 h post dose (Figure 6A). This concentration resulted in a 60% inhibition in cellular proliferation of CD44_high_ cells *in vitro* (Figure 1C). The calculated pharmacokinetic parameters (Table 2) were similar to those previously reported [26]. The dose (3 mg/kg) was well tolerated in all the animals in the pharmacokinetic study.

To evaluate the ability of INK128 to inhibit tumor progression, we conducted an efficacy study using CD44_high_ SK-Hep1 tumor bearing mice xenografts. Tumor bearing mice were treated with vehicle, sorafenib, INK128 or combination. A combination group was included as *in vitro* experiments suggested an additive/synergistic interaction between sorafenib and INK128. One-way analysis of variance shows significant difference between the treatment groups (p <0.0001). Pairwise comparisons between groups reveal significant differences between INK128-treated groups and vehicle control or sorafenib groups. INK128 prevented new tumor growth, and reduced tumor burden up to 30% compared to vehicle control or sorafenib alone (Figure 6B). Both vehicle and sorafenib treated groups showed an overall increase in tumor volume with treatment (Figure 6B). Although sorafenib resulted in approximately 15% inhibition in tumor growth compared to vehicle control, this inhibition was not significant. The efficacy of INK128 was comparable to studies in tumor models other than HCC [34–37].

We report here that ATP-competitive mTOR inhibitors exhibit improved anti-proliferative and anti-migratory effects in CD44-expressing, mesenchymal-like HCC cells. We further show that unlike sorafenib, INK128 is effective at reducing tumor burden in CD44 expressing HCC *in vivo*. As INK128 is currently being compared to sorafenib in a Phase I/II clinical trial in advanced or metastatic HCC (NCT02575339, clinicaltrials.gov), CD44 expression status may be an interesting biomarker to compare to efficacy within these patients. Collectively, our data suggests that response to INK128 correlates with CD44 expression status in HCC. Our findings propose a potential role of CD44 as a biomarker to predict a patient's response to ATP-competitive mTOR inhibitors such as INK128. ATP-competitive mTOR inhibitors should therefore be considered as promising therapeutic agents in CD44 positive HCC by regulating the aberrantly activated PI3K/AKT/mTOR pathway as well as CD44. These data suggest substantial clinical implications may be achievable for treating advanced HCC patients with limited treatment options.

## MATERIALS AND METHODS

### Cell culture

The human HCC cell lines PLC/PRF/5, HepG2, Huh7, SNU423, SNU449 and SK-Hep-1 were purchased from American Type Tissue Collection (Manassas, VA). PLC/PRF/5 and Huh7 cells were grown in MEM medium (Gibco) with 10% fetal bovine serum (Sigma). SNU423 and SNU449 cells were cultured in RPMI 1640 medium (Gibco) containing 10% fetal bovine serum (Sigma). All cell lines were recently authenticated at the Interdisciplinary Center for Biotechnology Research, University of Florida (Supplementary Table 1).

### Generation of Huh7 sorafenib resistant cell line

A sorafenib-resistant Huh7 cell line was established by long term exposure to incremental concentrations of sorafenib (LC Laboratories, USA) as reported [38]. Following six months of culture in 10 cm cell culture dishes, resistant cells were diluted out in 96 well plates and single cell clones were selected and scaled up to be used in further experiments.

### CD44 overexpression in CD44_low_ cells

An optimized DNA fragment for CD44 overexpression was designed and ordered from Invitrogen using GeneArt^®^ Gene Synthesis. The optimized DNA fragment was cloned using GeneArt^®^ Seamless Cloning technology into a p-EGFP-C1 vector graciously obtained from Matthew Wood's Lab. Huh7 and PLC/PRF/5 cells (CD44_low_) were transfected with 1500 ng of either CD44 vector or empty vector using Lipofectamine 3000 (Invitrogen). Forty-eight h after transfection, cells were selected using 150 μg/ml hygromycin (Life Technologies). Western blotting was used to confirm successful transfection and expression of CD44.

### RNA extraction and quantitative RT-PCR

Total RNA was isolated from ATP-competitive mTOR inhibitor treated cells using miRNeasy^®^ Mini kit (Qiagen). cDNA was synthesized according to the manufacturer's protocol (Invitrogen). Five hundred ng of total RNA was used to synthesize cDNA using random primers. cDNA was analyzed for gene expression using gene specific primers (IDT) and the Express SYBR^®^ GreenER qPCR super mix (Invitrogen). Data were normalized to 18S rRNA and the relative expression of genes was presented using the comparative C_T_ method. The following primers were used; CD44: 5′-TGCAGTT TGCATTGCAGTC-3′ (forward) and 5′-CATTGCCAC TGTTGATCACTAG-3′ (reverse), Vimentin: 5′-CAGC TAACCAACGACAAAGCC-3′ (forward) and 5′-ATCCT GTCTGAAAGATTGCAGGG-3′ (reverse), 18S: 5′-GT AACCCGTTGAACCCCATT-3′(forward) and 5′-CCAT CCAATCGGTAGTAGCG-3′(reverse)

### Immunoblotting

Total protein from PLC/PRF/5, Huh7, sorafenib resistant Huh7, SNU423 and SNU449 were extracted with radio immunoprecipitation assay (RIPA) buffer. Cell protein lysates (20–30 μg) were separated on NuPAGE 4–12% Bis-Tris gels (Novex) electrophoretically and transferred to polyvinylidene difluoride membranes (Roche). Membranes were blocked for 1 h with 5% Bovine Serum Albumin in Tris-buffered saline containing 0.05% Tween 20 and incubated overnight with primary antibody. The following primary antibodies were used: anti-CD44 (Cell signaling, #3578), anti-vimentin (Abcam, ab92547), anti-total S6K (Cell signaling, # 2708), anti-phosphorylated S6K (Cell signaling, #9234), anti-total 4EBP-1 (Cell signaling, #9644), anti-phosphorylated 4EBP-1(Cell signaling, #2855) and anti-PARP (Cell signaling # 9532) Anti-GAPDH (Santa Cruz Biotechnology, sc-32233) and anti-β actin (Cell Signaling, 4970S) antibodies were used as loading controls. A secondary anti-rabbit (Cell Signaling) or anti-mouse immunoglobulin G (IgG) antibody peroxidase conjugate (GE Healthcare) was detected using ECL Western Blotting Analysis System (Amersham Biosciences).

### Cell proliferation assay

PLC/PRF/5, Huh7, sorafenib resistant Huh7, SNU423 and SNU449 cells were seeded at 3,000 cells per well in 96-well culture plates one day before treatment with mTOR inhibitors (dissolved in 1% DMSO) or sorafenib. Cell viability was determined by the WST-1 reagent (Roche) according to the manufacturer's recommendations. Absorbance at 450 nm was measured 48 or 72 h after treatment of rapamycin, everolimus, PP242 (Sigma Chemical Company), INK128 (Intellikine LLC, La Jolla, CA) or 1% DMSO vehicle control. All experiments were performed at least in triplicate.

### Cell growth and doubling time

Huh7 and PLC/PRF/5 cells stably expressing empty or CD44 vectors were seeded at 5,000 cells per well in a 24-well culture plate. The cells were trypsinized and counted daily using Trypan Blue (Life Technologies).

### Wound healing assay

SNU423 cells were treated with either INK128 or everolimus at 500 nM or vehicle control (1% DMSO). At 24 h after treatment or transfection, 70 μl of the treated cells (3 × 10^5^ cells/mL) was placed into each well of ibidi culture-insert (ibidi, LLC). After an overnight incubation, the culture insert was removed to create a cell-free gap in a monolayer of the cells. The gap closure area was photographed and analyzed by Tscratch software [39]. Wound healing assay was further performed using Huh7 and PLC/PRF/5 cells to determine the effects of CD44 overexpression of migration. The percentage of the gap closure area based on the initial point at time zero was calculated from at least two independent experiments.

### Sorafenib and INK128 interaction

The nature of interaction between sorafenib and INK128 was assessed *in vitro* in CD44_high_ SNU423 and SNU449, as well as CD44_low_ Huh7 cells. IC_50_ values for both drugs in each cell line were determined and used to maintain a fixed molar ratio in combination studies. Cells were treated with single agent separately and in combination for 72 h and analyzed using WST1 reagent. Combination index values were determined using the median effect method described by Chou and Talalay [40].

### INK128 pharmacokinetic studies

All animal experiments were carried out under protocols approved by the Institutional Laboratory Animal Care and Use Committee at the Ohio State University. Imprinting control region (ICR) female mice (6 weeks) were obtained from Charles Rivers (Wilmington, MA, USA). Mice were acclimated in the animal housing facility for one week before the start of the study. After overnight fasting, INK128 (3 mg/kg) was administered by oral gavage (PO) in 10% DMSO. At different time points, mice were euthanized by carbon dioxide asphyxiation, and blood was immediately collected by cardiac puncture into lithium heparinized tubes (BD Microtainer, Becton, Dickson and Company, NJ, USA). Blood samples were centrifuged at 6000 × g for 5 min at 4°C. Plasma was collected and kept on dry ice before being stored at −80°C until analysis.

### Sample preparation and analysis

Plasma samples were thawed on ice. Blank mouse plasma was used for standard curves. Plasma aliquots (100 ul) were spiked with hesperetin (10 ul, 5000 ng/ml) as an internal standard and mixed by vortexing. Drugs were extracted with simple protein precipitation using cold acetonitrile, and the mixture was centrifuged at 13000 × g for 10 min. The supernatant was collected and dried under nitrogen. Dried samples were reconstituted with 200 ul 1% formic acid. Samples were analyzed using liquid chromatography–tandem mass spectrometry (LC-MS/MS) system consisting of Thermo Accela UHPLC pump, Thermo PAL autosampler, and Thermo TSQ Discovery triple quadruple mass spectrometer (Thermo, Waltham, MA, USA). Chromatographic separation was done using an Agilent Zobrax Extend C-18 column. Linear gradient elution was used at a flow rate of 400 ul/min, with a mobile phase of 0.5% acetonitrile and 95% acetonitrile, modified with 0.2% formic acid. Non-compartmental analysis of the pharmacokinetic parameters as well as compartmental model fitting of the data was carried using Phoenix^®^ 7.0 (Certara, New Jersey, USA).

### Establishing SK-Hep1 murine xenografts

Immunodeficient athymic nude male mice (8 weeks) were obtained from Charles River laboratories and kept in the University's animal housing facility. SK-Hep1 cells were prepared from a low-passage, logarithmically growing population of cells. SK-Hep1 (3 × 10^6^ cells) were suspended in 150 ul of Matrigel (Corning, NY, USA) and injected subcutaneously into the right flank of the mice. Three weeks after implantation, tumor sizes were measured using a Fisherbrand™ Traceable™ Digital Caliper (Thermo), the animals were randomized based on tumor size, and treatment was initiated. Tumor size measurements were taken twice a week throughout the treatment period.

### Xenograft drug treatments

Athymic nude mice bearing SK-Hep1 tumors were randomized into four groups (*n* = 10 per group); vehicle, sorafenib, INK128 and combination. Sorafenib (10 mg/kg) was prepared as a 4× stock solution in cremophor EL:ethanol (50:50), and diluted using water on the day of the treatment [41]. Vehicle and sorafenib groups received their respective treatments every day via oral gavage. INK128 (3 mg/kg) was prepared in 10% DMSO and dosed via oral gavage every other day. The combination group received a daily dose of sorafenib (10 mg/kg) along with INK128 (3 mg/kg) every other day. Mice were monitored every day, and body weight and tumor size measurements were recorded twice a week throughout the treatment period. Tumor volume was calculated using the equation V= ½ × L × W^2^. Treatment was continued for 24 days after which the animals were euthanized. Tumor growth is represented as percentage change from baseline tumor volume.

### Statistical analysis

The wound healing assay data between three different treatments were compared using ANOVA. The effect of CD44 overexpression on wound healing and proliferation were analyzed using a two sample *t*-test. All error bars represent the standard deviation of the mean. A *p*-value < 0.05 was considered significant. Concentration response curves from the proliferation experiments were fitted using Sigma Plot 13.0 (Systat Software Inc. Chicago, USA). Sorafenib and INK128 IC_50_ values were calculated using the four parameter Hill equation. Calcusyn software (Biosoft, Cambridge, UK) was used to calculate combination indices. Values CI < 1 indicate synergy, CI > 1 indicate antagonism and CI = 1 indicate an additive effect. Tumor growth studies were analyzed using ANOVA as well as pairwise *t*-test comparisons between the treatment groups.

## SUPPLEMENTARY MATERIALS FIGURES AND TABLES


