# Supplementary material for: CD44 positive and sorafenib insensitive hepatocellular carcinomas respond to the ATP-competitive mTOR inhibitor INK128
Source: Oncotarget. 2018 May 25;9(40):26032–45. doi: 10.18632/oncotarget.25430 (PMC5995255; doi:10.18632/oncotarget.25430)
Supplement: Supplementary file 1 [file oncotarget-09-26032-s001.pdf]

CD44 positive and sorafenib insensitive hepatocellular carcinomas respond to the ATP-competitive mTOR inhibitor INK128

SUPPLEMENTARY MATERIALS

Supplementary Table 1: Cell line authentication

Name	Origin	Certification institution	Tested method	DNA profile or characteristics
SNU-423	Human liver cancer	ICBR at UFL	STR	D5S818: 10, D13S317: 10, 13, D7S820: 12, D16S539: 9, vWA: 15, TH01: 9, AMEL: X, Y, TPOX: 8, CSF1PO: 11, 12, D21S11: 30
SNU-449	Human liver cancer	ICBR at UFL	STR	D5S818: 10, D13S317: 9, D7S820: 8, 13, D16S539: 9, vWA: 14, 16, TH01: 6, 9, AMEL: X, TPOX: 11, CSF1PO: 11, D21S11: 29, 31
HepG2	Human liver cancer	ICBR at UFL	STR	D5S818: 11, 12, D13S317: 9, 13, D7S820: 10, D16S539: 12, 13, vWA: 17, TH01: 9, AMEL: X, Y, TPOX: 8, 9, CSF1PO: 10, 11, D21S11: 29, 31
PLC/PRF/5	Human liver cancer	ICBR at UF	STR	D5S818: 12, D13S317: 11, 12 D7S820: 9, 11, D16S539: 13 vWA: 15, 16, TH01: 7, 8, AMEL: X, TPOX: 8, CSF1PO: 10, D21S11: 30, 33.2
SK-Hep-1	Human liver cancer	ICBR at UF	STR	D5S818: 10, 13, D13S317: 8, 12 D7S820: 8, 11, D16S539: 12, vWA: 14, 17, TH01: 7, 9 AMEL: X, TPOX: 9 CSF1PO:11, 12, D21S11: 29, 31
HuH-7	Human liver cancer	ICBR at UF	STR	D5S818: 12, D13S317: 10, 11, D7S820: 11, D16S539: 10, vWA: 16, 18, TH01: 7, AMEL: X, TPOX: 8, 11 CSF1PO:11, D21S11: 30

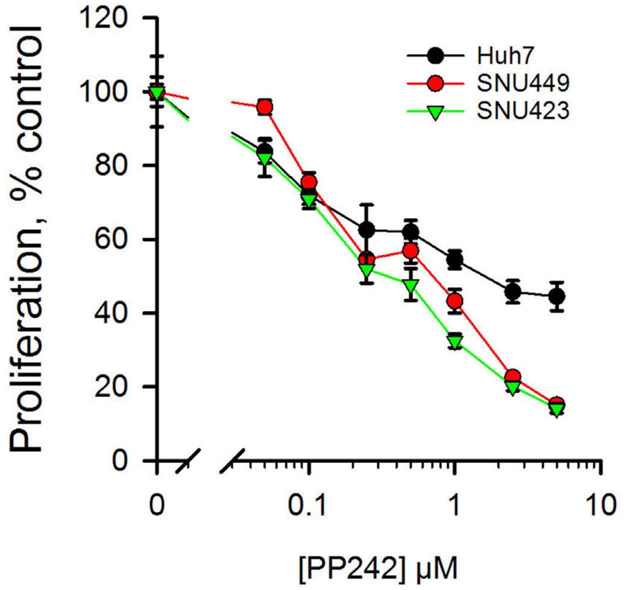

Supplementary Figure 1: PP242 shows enhanced anti-proliferative activity in CD44$_{high}$ HCC cells. Three different HCC cell lines (CD44$_{high}$, SNU423 and SNU449) or CD44$_{low}$ (Huh7) were treated with the ATP-competitive mTOR inhibitor PP242 for 48 h. Cell proliferation was determined by a WST-1 assay. Single replicate from at least duplicate experiments. Mean ± S.D.

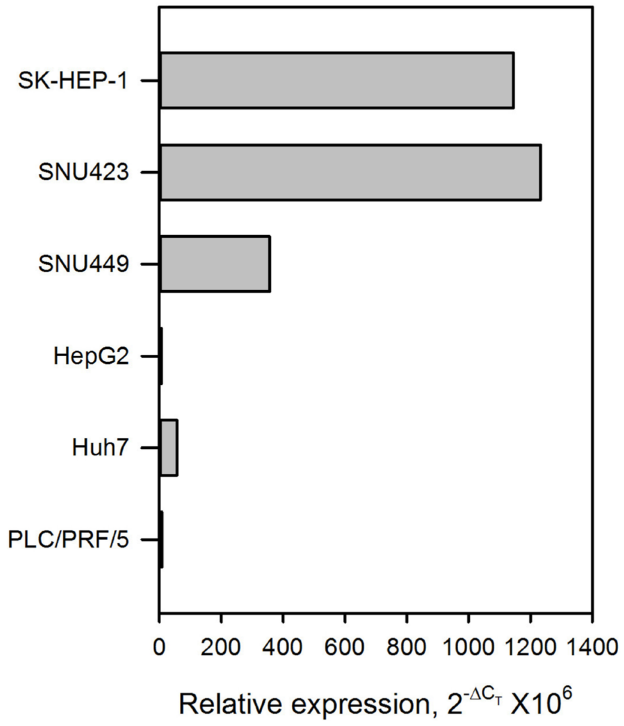

Supplementary Figure 2: CD44 mRNA expression in HCC cell lines. The expression of CD44 was determined by qPCR in 6 HCC cell lines. Data are normalized to 18S rRNA.

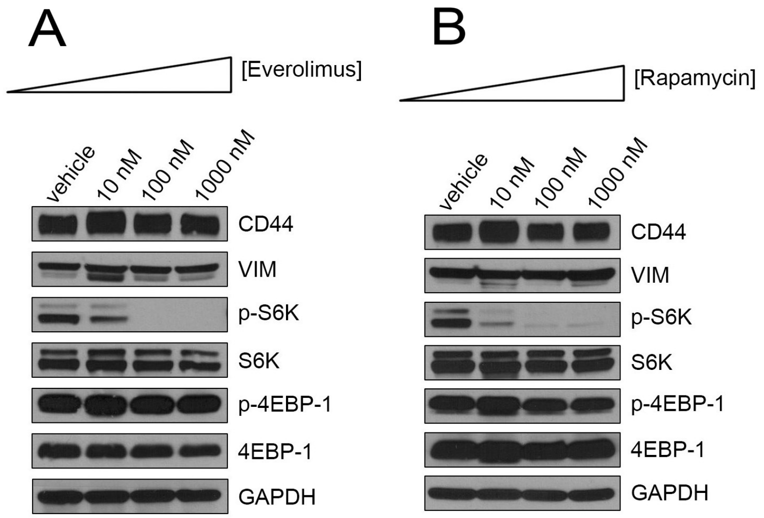

Supplementary Figure 3: Rapalogs do not reduce CD44 and Vimentin and downstream effector of mTOR pathway. $CD44_{high}$ SNU449 cells were treated with increasing concentrations of (**A**) everolimus or rapamycin (**B**). CD44, vimentin and downstream effectors of mTOR pathway were evaluated by immunoblotting.

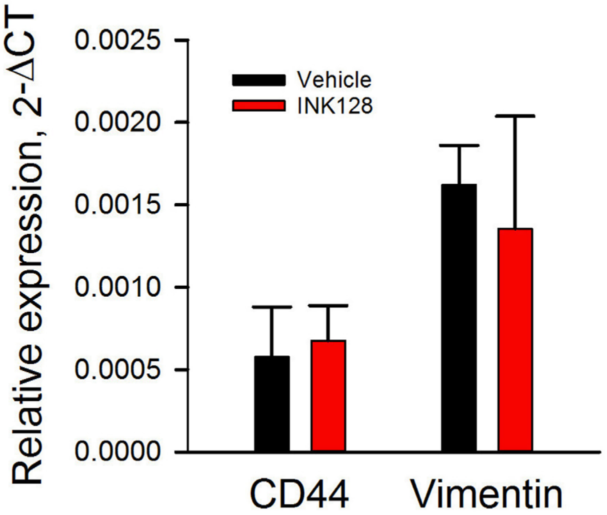

Supplementary Figure 4: No change in CD44 or Vimentin mRNA upon treatment with INK128. CD44 and vimentin mRNA levels were measured by qPCR in SNU423 cells treated with 500 nM INK128 48 h.

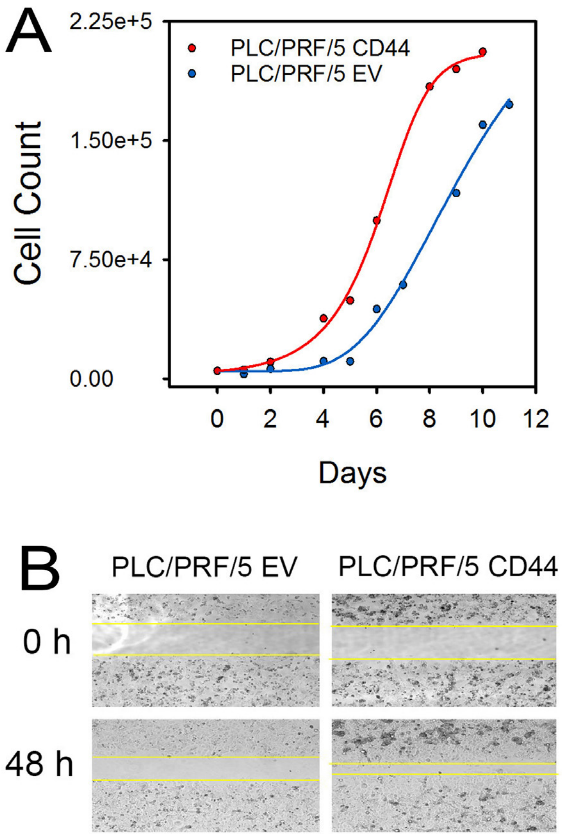

Supplementary Figure 5: CD44 overexpressing PLC/PRF/5 cells have a more aggressive phenotype and improved response to INK128. PLC/PRF/5 cells were stably transfected with CD44 expressing vector or empty vector (EV) control. (**A**) Cell growth curves of PLC/PRF/5_{CD44} and PLC/PRF/5_{EV} cells. (**B**) *In vitro* migration of PLC/PRF/5_{CD44} and PLC/PRF/5_{EV} cells. Dashed lines represent the initial starting point of the assay at time 0.
